# Gender and zoonotic pathogen exposure pathways in a resource-limited community, Mpumalanga, South Africa: A qualitative analysis

**DOI:** 10.1371/journal.pgph.0001167

**Published:** 2023-06-05

**Authors:** Pallavi Oruganti, Elisabeth Root, Violet Ndlovu, Philemon Mbhungele, Ilana Van Wyk, Amanda M. Berrian

**Affiliations:** 1 Department of Veterinary Preventive Medicine, The Ohio State University College of Veterinary Medicine, Columbus, Ohio, United States of America; 2 College of Public Health, The Ohio State University, Columbus, Ohio, United States of America; 3 University of Pretoria, Pretoria, South Africa; University of Bremen: Universitat Bremen, GERMANY

## Abstract

The Mnisi community is a livestock-dependent community neighboring the Great Limpopo Transfrontier Conservation Area in South Africa. Here, zoonotic pathogens contribute to as many as 77% of cases of acute febrile illness. Previous gender-disaggregated analysis in the community has shown that men and women have different risks of zoonotic illness, suggesting that exposure routes for zoonotic infections should be further explored to inform gender-sensitive risk mitigation strategies. Using a One Health approach and ethnographic methodology, we examined interactions between community residents, domestic animals, and the built and natural environment to investigate potential exposure pathways for zoonotic infections from a gendered perspective. We combined data from direct household observations and focus group discussions on previously identified gendered tasks such as domestic animal care, water collection, and food preparation, and how and by whom these tasks were performed. We noted gender differences for household tasks, animal care duties, and environmental exposure. Both men and women access grazing land but for different tasks (water collection—females, cattle grazing—males), and both men and women experience more time in the bush in recent years due to decreased water availability. From observations, it was noted that men wore covered protective work clothes (such as long trousers and closed-toe shoes) more commonly than women did; women did not often wear these for household duties including water collection in the bush. We recommend that these gender-typed roles serve as critical control points for zoonotic pathogen exposure. For example, tick-bite exposure prevention should be directed at both men and women based on their daily activities, but prevention in men should target exposure from cattle and prevention in women should focus on personal protective measures during water and firewood collection. These findings can contribute to a more detailed understanding of the role of human behavior and critical control points for zoonotic disease—a significant contributor to acute febrile illness in this rural, resource-limited setting.

## Introduction

Around the world, the impact of infectious disease is disproportionately high in low-resource settings [1] and further compounded by daily livelihood activities which can be impacted by sociodemographic factors, such as age and gender [2]. Neglected zoonotic diseases are closely associated with poverty and they disproportionately affect poor and neglected populations who are more at risk of contracting many zoonoses [3]. Gender, referring to characteristics of women, men, girls and boys that are socially constructed [4], is related to differential pathogen exposure because of inherent gender norms influencing daily activities. Men and women have differing occupational exposures, division of labor and family care duties, domestic household chores, and exposures to livestock [2]. Gender disparities in exposure and mortality have been noted in infectious disease outbreaks including COVID-19, Ebola, and avian influenza [2, 5, 6]. For the recent COVID pandemic, men have had higher mortality worldwide likely due to several gender-based societal roles and biological factors unique to women [6, 7]. Socially constructed, societal beliefs about masculinity and femininity can also impact health outcomes. In South Africa, masculinity and cultural norms are major influencers of health-seeking behavior in men, including the underutilization or delayed use of health care services [8].

In rural societies, where local culture and traditions shape daily life responsibilities, tasks are often assigned to women and men based on traditional gender roles, defined as those behaviors and responsibilities that a culture or society considers appropriate for men, women, boys and girls [9]. Gender roles are a key determinant of the distribution of resources and responsibilities between men and women. Gendered differences in livestock-caretaking duties and exposure to wild animals are primary factors influencing gender differences in infectious disease exposure, especially of zoonotic origin. Zoonotic diseases can be acquired from either domestic or wild animals or through environmental and vector exposure. Occupations that involve close contact with wild animals or their habitats, such as hunting, forestry, and mining are usually male dominated. Occupations on large commercial farms also tend to be male dominated. In contrast, work that involves the care and feeding of animals kept close to home or in small backyard farms is often performed by women and children [2].

A web of interconnected circumstances contributes to defining exposure for men and women to zoonotic pathogens [10]. Understanding the interaction between gender roles and infectious disease can lead to important insights into transmission patterns and strategies for prevention and control, thereby reducing disease incidence in vulnerable communities [11]. Taking a One Health approach to zoonotic disease research, and specifically gender differences influencing pathogen exposure pathways, can be an effective method to address these variable factors. One Health is the conceptual framework integrating human, animal, plant and environmental health [12, 13]. To address the complex socio-cultural and gendered factors influencing zoonotic pathogen exposure outside of animal health and biological drivers of disease, One Health approaches that integrate social science, participatory, and ethnographic methods should be used [14–19]. This approach has been effectively applied to better understand the structural factors contributing to zoonotic disease in rural settings, including studies on trypanosomiasis, Ebola, and Rift Valley Fever in Africa [20].

In southern Africa, a unique context exists to evaluate the social drivers of ecological changes and infectious disease, in part due to the establishment of Transfrontier Conservation Areas (TFCAs) [21]. TFCA’s are conservation areas in southern Africa that aim to preserve the native ecology and landscape of areas that cross country borders. They also preserve natural animal migration patterns and protect from human encroachment [21]. The Mnisi community in Mpumalanga, South Africa encompasses an area that is part of the originally established Greater Limpopo TFCA and the Kruger to Canyons Biosphere Region [22]. The Mnisi Community Programme (MCP), established in 2008 in collaboration with the Mnisi community and the University of Pretoria, has focused on addressing community health at the wildlife-livestock interface including human and veterinary medical care, ecosystem health and land use, and disease ecology and zoonotic disease by providing veterinary services, education programs to the community, and scientific research programs [23].

Residents of the Mnisi community co-exist with livestock and a biodiverse, dynamic wildlife population. Due to these landscape and lifestyle factors, residents are considered “high risk” for zoonotic pathogen exposure [24, 25]. More than three-quarters of adults presenting to local health clinics with acute febrile illness had evidence of infection or exposure to zoonotic pathogens, namely *Rickettsia* spp., *Leptospira* spp., *Bartonella* spp., and *Coxiella burnetii* [26]. For the most common zoonosis identified, spotted fever group rickettsiosis, gender was found to be a significant risk factor; odds of seropositivity were eight times higher in females than males [27]. A follow-up study in the community found daily livelihood tasks (e.g., water collection, food preparation) and livestock care (e.g., birthing, slaughtering) were highly gendered, indicating differential risks to men and women of zoonotic, foodborne, and water-borne diseases [28].

**Fig 1:**
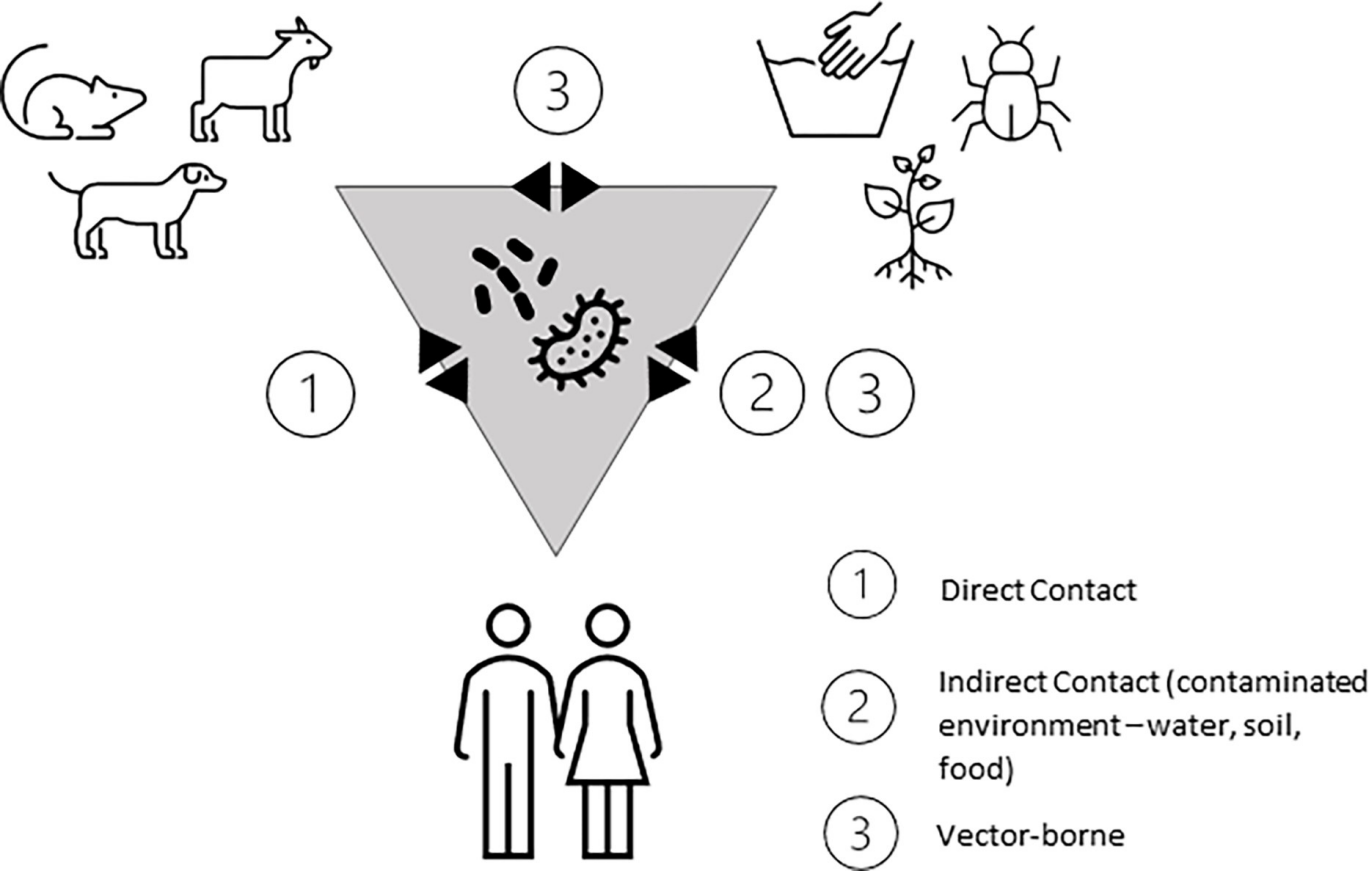
Exposure pathways of priority zoonoses associated with acute febrile illness (leptospirosis, rickettsiosis, and Q fever) in the Mnisi community, Mpumalanga, South Africa.

Given the importance of certain zoonotic pathogens for acute febrile illness in the community, the significance of gender as a risk factor for disease and potential exposure pathways, this study aimed to provide a more in-depth qualitative view of gendered tasks in this community to contextualize these gender roles in the greater cultural and environmental landscape. Components of gender analysis and gendered frameworks for studying infectious neglected tropical diseases were also integrated to ensure gender was prioritized in the analysis within the One Health approach [29, 30]. We focus our study on the exposure pathways of certain zoonotic diseases, namely spotted fever group rickettsiosis, Q fever, and leptospirosis, as we have evidence of these diseases in adult members of the community. Additionally, these diseases are representative of a variety of probable transmission routes of zoonoses, including direct contact, indirect contact via contaminated environment (food, water, air, soil), and vector-borne. Fig 1 visualizes the transmission dynamics of these select pathogens in the human-animal-environment interface.

**Fig 2:**
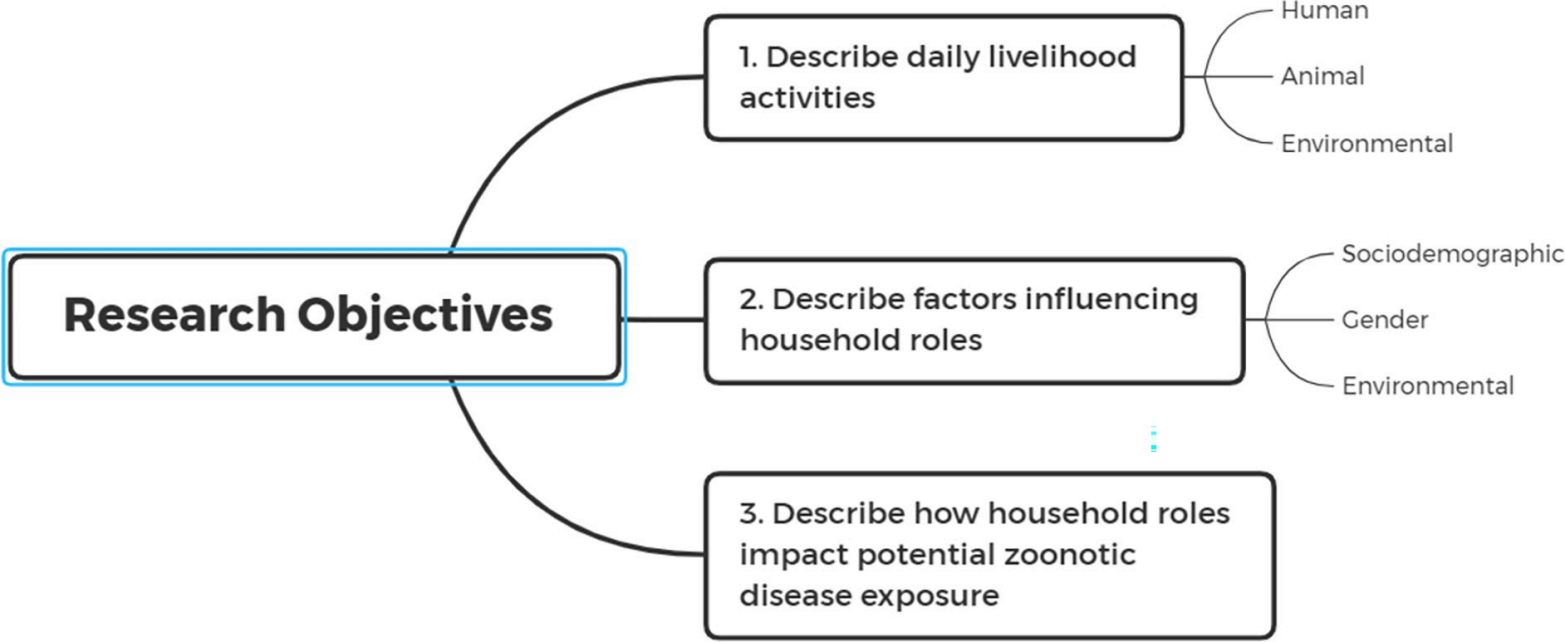
Primary research objectives.

Building on previous research in the study area on gender and infectious disease, the primary objectives of this study were to further describe gendered livelihood activities, how socio-cultural factors influence these roles and, in turn, how these roles may relate to exposure pathways of certain zoonoses relevant to the community [24] (Fig 2). We can categorize the activities based on these pathways and further describe how these activities are performed to identify specific critical control points for the prevention of acute febrile illness of zoonotic origin.

## Methods

### Study area

**Fig 3:**
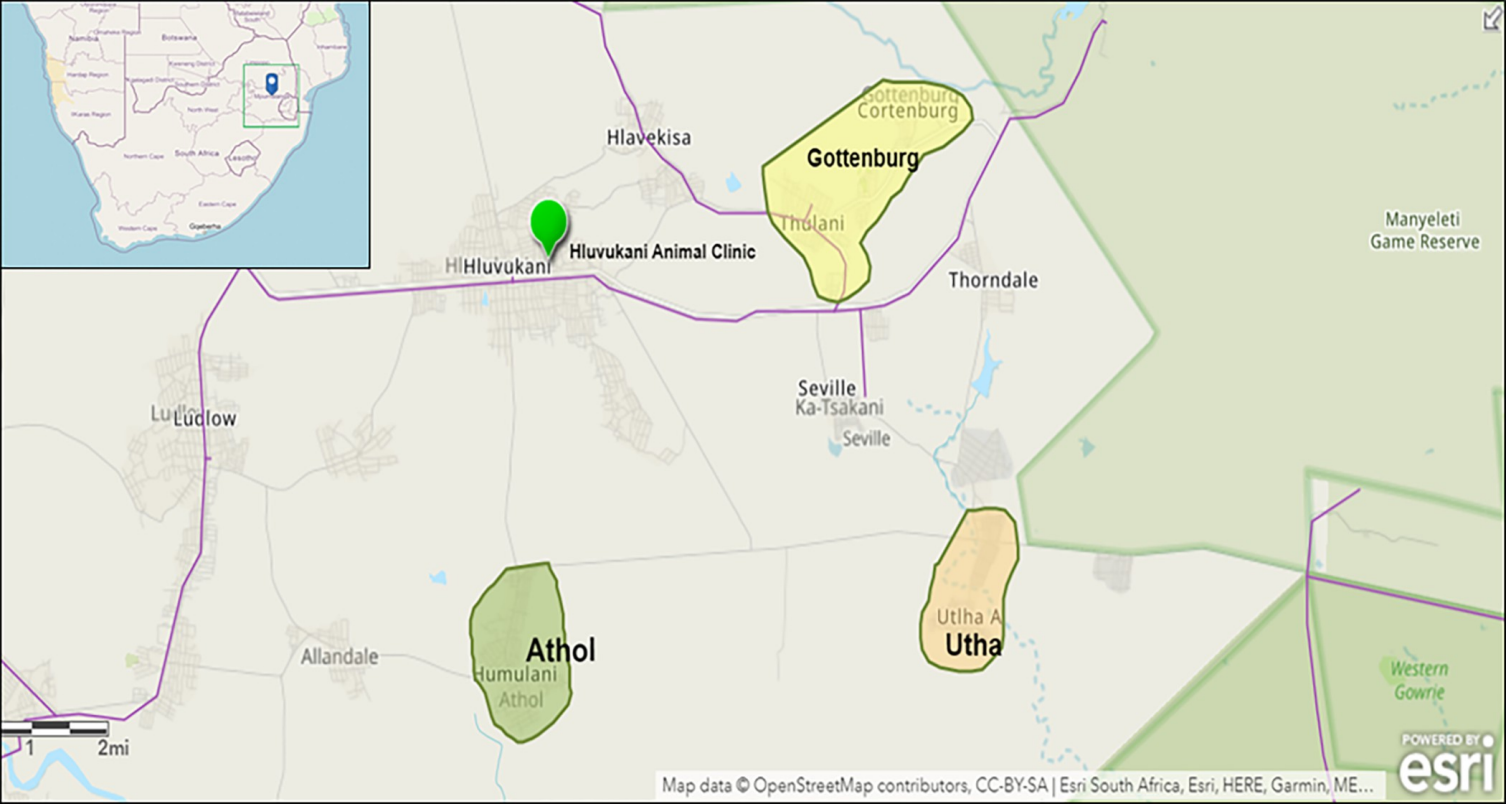
Map of study area. The Fig 3 map was created with OpenStreetMap, OpenStreetMap is open data, licensed under the Open Data Commons Open Database License (ODbL) by the OpenStreetMap Foundation (OSMF) and is licensed under the Creative Commons Attribution-ShareAlike 2.0 license (CC BY-SA 2.0, https://www.openstreetmap.org/copyright).

This study was conducted in Bushbuckridge Local Municipality, Mpumalanga Province, South Africa. Fieldwork was conducted in the Mnisi community, within the area of the MCP of the University of Pretoria. The Mnisi study area consists of approximately 9,000 households with a population over 40,000 people [31]. The area encompasses communities at the human-wildlife-livestock interface, as they are bordered by both private and provincial game reserves and natural savannah areas. The community is adjacent to the Great Limpopo TFCA jointly managed by South Africa, Mozambique, and Zimbabwe, and the Kruger-to-Canyons UNESCO Biosphere Region of conservation and sustainable socioeconomic development. The TFCA bordering the community situates the study area at a wildlife-human-livestock interface. The community is semi-pastoralist, largely livestock-dependent, and household ownership of domestic animals such as cattle, goats, chickens, and dogs are common [24]. Three villages from the Mnisi community were chosen for the study: Utha, Athol, and Gottenburg (Fig 3) [32]. These villages were selected based on their prior involvement with the research team and the desire for continued, longitudinal community engagement.

### Framework and research questions

This study drew conceptually from frameworks of One Health and ecosocial theory. We used a One Health approach, integrating components of human health and behavior, domestic animals, wildlife, and other environmental and ecosystem considerations into the study design. As we used qualitative ethnographic methods, ecosocial and social-ecological models of infectious disease served as guiding theories for the study approach, research questions, and methods [33]. These theories propose that disease patterns in a population are shaped by broader societal and ecological contexts, including gender, and the complex interplay between them; thus, including One Health and ecosocial better addresses health promotion, disease prevention, and well-being at the community level [34, 35]. Ecosocial theory calls us to consider the social state and the consequential embodiment of thoses social and environmental states in our analysis of disease causation and population health. These social states, inevitably, include gender and its impact on health outcomes [36]. Components of gender analysis and gendered frameworks for studying infectious neglected tropical diseases were also integrated to ensure gender was prioritized in the analysis of potential exposures within the One Health approach [29, 30].

The study design and research questions were based on understanding the interactions between humans, animals, and the environment, and the transmission routes for zoonotic pathogens prevalent in the community associated with acute febrile illness in adults. The study design seeks to incorporate participatory methods and considerations of gender in studies of infectious zoonotic disease and animal health as social and cultural factors, including gender, can influence exposure and, ultimately, health outcomes [11, 15, 31]. Focus group questions and the observation protocol were developed based on a One Health framework, with questions and observations focusing on human, domestic animal, wildlife, and environmental interactions and considerations of daily routines and household roles [34, 35].

### Ethics

The study was reviewed by The Ohio State University Office of Responsible Research Practices and determined to be exempt from Institutional Review Board review (ref no.: 2019E0494). Additionally, the study was reviewed and approved by the University of Pretoria Research Ethics Committee (ref no.: REC089-19). Community tribal authority approval was also sought at each participating village prior to commencing the study. During recruitment, Environmental Monitors (EMs), local community members and employees of the Kruger to Canyons biosphere, translated a consent script explaining the study objectives to prospective participants and emphasized the voluntary and anonymous nature of participation; verbal informed consent was received before further participation from the head of household present at the household observation and from each focus group participant.

### Participant selection

We used a qualitative study design, triangulating data collected through ethnographic household observations and focus group discussions (FGD) [37–39]. Households from each of the three villages were selected through random sampling to participate in the observations and FGDs. Additional criteria for participation included animal ownership or visible proximity of households to domestic animals. Recruitment for household observations occurred during weekday daytime hours (8am– 5pm). Focus group participants were also recruited randomly and by word of mouth (snowball sampling) from the community the day before the focus group was to be held and were instructed to meet at a central but private area in the community. Upon receiving their consent, participants were enrolled in the study.

### Data collection

Data collection occurred over four weeks in July 2019. We conducted four household observations per village and two focus groups per village, one with women and one with men. Household observations were limited to adult (aged 18+) members when verbal informed consent could be received. Information pertaining to children was obtained from adult study participants during observations and FGDs. We aimed to enroll 5–7 participants in each focus group to achieve a representative group but also to maintain clarity and flow of the group discussion [37]. Focus groups were separated by gender to ascertain specific information and attitudes about gender roles and provide a comfort level to participants in addressing gender-related topics. Observations lasted about four hours each. Observations were conducted during varying time periods during the day in both the morning and afternoon to be more representative of livelihood activities in the household throughout the day. Nighttime observations were not possible due to restrictions for traveling to/from the community. This yielded a total of 12 observations and six focus groups discussions. Sample size was based on the number of interviews and observations needed to achieve data saturation and the timeline of the fieldwork, which was determined by the availability of the study team and funding period [40].

### Observations

Verbal consent was obtained from the head of household; that individual was interviewed to determine household-level demographic data (e.g., age, occupation, employment, number/type of owned animals). Observations were conducted with the individuals present at the home during the observation time-period. Field notes were taken during the observations in addition to demographic information that was recorded for each household being observed. An observation log was created to document the demographic information of the household, observed tasks, person performing the task, the time of day, total time spent on the task, contacts with other people or animals during the task, and any other comments on the nature of the action or interaction. With permission, photos were taken during observations at each household of the environment, structure, and of certain behaviors. All household observations were attended by 3–4 members of the study team, which included two community-based Environmental Monitors who served as translators for the consent process, the initial demographic interview, as well as to clarify observations.

### Focus groups

Each FGD was moderated by one trained facilitator and two Environmental Monitors who served as translators. The facilitator used a semi-structured questionnaire (S1 File) to lead the discussion. The questionnaire was developed *a priori*, then updated and informed by the household observations. Question categories included demographic information, daily routine, animal care activities, hygiene, outdoor activities and disease prevention, with additional room for topics brought up during discussion. Focus group participants were not individually identified so their anonymity could be maintained. Discussions were audio-recorded, translated to English from Shangaan in real time, and then transcribed in English. The transcriptions were verified with the recordings by the Environmental Monitors, and field notes of participant responses during the FGDs were also taken.

#### Data analysis

**Fig 4:**
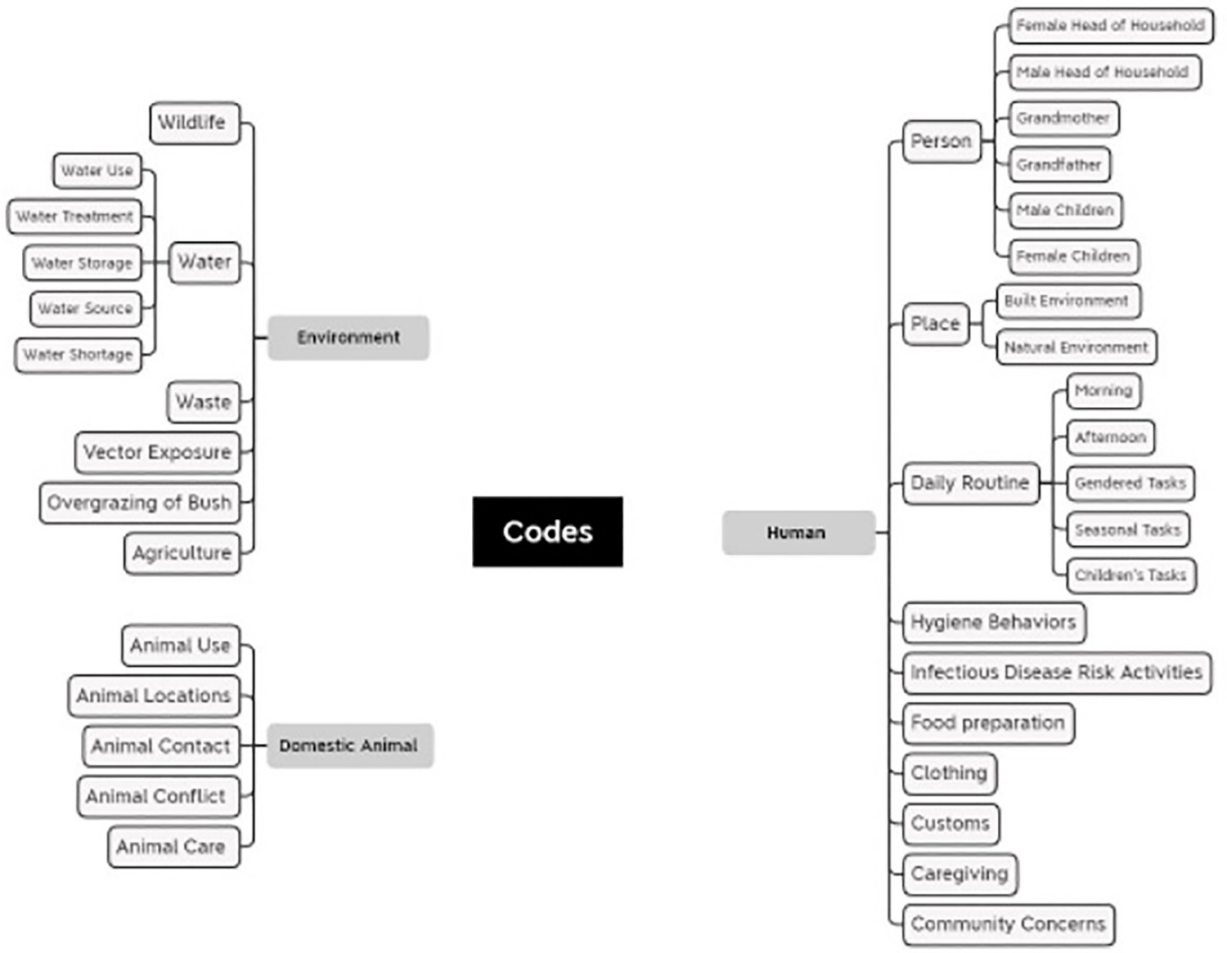
Final code and subtheme schematic.

Demographic data of the participants were summarized using descriptive statistics. Analysis of the qualitative data focused on thematic coding of both observations and FGDs. Transcripts and observation notes were uploaded into the NVivo 12 Plus qualitative analysis software. In NVivo, the data were reviewed first using a deductive coding method, focusing on themes seen within the One Health framework and daily livelihood activities. Coding was conducted based on principles of fundamental grounded theory [37]. Data were reviewed in an iterative and recursive manner by the researchers to identify novel codes or themes in multiple reviews, first with an initial coding of the data, then a line-by-line coding, and finally with further categorization of the emergent codes and themes [41]. Codes and themes were reviewed and grouped based on similarity or intent, resulting in the final code schematic (Fig 4). Data were triangulated between the observations and focus groups to compare and contextualize the data from each data source type. Results for focus group data was summarized across all the focus groups, and results for observations were summarized across all households observed. Field notes were used to contextualize observations and FGDs and note sentiments, other conversations or situational circumstances that elucidate the data collected [42]. After initial coding of the data was completed for both focus groups and observations, the data were triangulated to assess common themes that could be combined. Data between focus groups and observations were triangulated and codes were compared in later stages of coding and further iterations of coding. Where a specific action was observed in the observations, we examined specific parts of the focus groups that included discussions about these topics and provided further context. The demographic and focus group data are stored and available for open access in the Dryad database [43].

## Results

### Demographic and descriptive data

#### Household observations

A total of 12 household observations (four in each of the three villages) were completed fora total of 51.5 hours of observation. Households in the study area consisted of multi-generational and extended family home compounds, common in rural South African communities [31]. Descriptive statistics of the households observed are listed in Table 1. About half of adults (48%, 30/63) in the observed households were unemployed or students (28%, 17/63), with other varying professions noted including engineer, field guide, housekeeper, nurse, tracker, teacher, and Expanded Public Works Programs employee.

**Table 1:** Demographic summary from household observation participants (n = 12).

Village		Household Number	House Demographic Summary		Value
*Athol*		A1	Length of observation (hours)		2
			Number of Adults / Children		5 / 3
			Total Individual Animals		7
		A2	Length of observation (hours)		2
			Number of Adults / Children		4 / 4
			Total Individual Animals		0
		A3	Length of observation (hours)		9
			Number of Adults / Children		6 / 6
			Total Individual Animals		24
		A4	Length of observation (hours)		4.5
			Number of Adults / Children		11 / 4
			Total Individual Animals		26
*Utha*		U5	Length of observation (hours)		4
			Number of Adults / Children		3 / 2
			Total Individual Animals		67
		U6	Length of observation (hours)		4
			Number of Adults / Children		2 / 4
			Total Individual Animals		0
		U7	Length of observation (hours)		4
			Number of Adults / Children		4 / 2
			Total Individual Animals		32
		U8	Length of observation (hours)		4
			Number of Adults / Children		3 / 2
			Total Individual Animals		27
*Gottenburg*		G9	Length of observation (hours)		3
			Number of Adults / Children		8 / 5
			Total Individual Animals		27
		G10	Length of observation (hours)		4.5
			Number of Adults / Children		4 / 2
			Total Individual Animals		63
		G11	Length of observation (hours)		5
			Number of Adults / Children		4 / 1
			Total Individual Animals		30
		G12	Length of observation (hours)		5.5
			Number of Adults / Children		9 / 3
			Total Individual Animals		56
**Household Composition**				**Value**	**SD**
	Total Individuals in all Observation Households			100	
	Total Children in Observed Households			37	
	*Male*			14	
	*Female*			23	
	Total Adults in Observed Households			63	
	*Male*			19	
	*Female*			44	
	Mean Number of Members per Household			8.33	3.63
	Mean Number of Adults per Household			5.25	2.73
	Mean Number of Children per Household			3.08	1.44
**Employment—Adults**					
	Unemployed/pensioner			30	
	Student			17	
	Other			16	
**Highest Level of Education Completed—Adults**					
	No formal education			16	
	Grade 3			1	
	Grade 7			7	
	Grade 11			3	
	Grade 12			34	
	College			8	
**Mean animals per species**				***Mean***	**SD**
Chicken				13.9	5.43
Pig				6.333333	4.03
Goat				11.8	6.3
Cattle				14.875	10.78
Dog				2	1.73
Cat				3	0
**Mean number of species owned per household (of those with animals)**					
				3.2	1.3
**Mean number of individual animals (total) per household (of those owning animals)**					
				35.9	19.4

#### Focus group discussions

Six single-gender focus groups were conducted (one male group and one female group per village). The size of the focus groups ranged from 4–10 people (mean = 7). Most participants (59%, 26/44) were unemployed outside the home, with other employment/income sources including community caregivers, self-employed, construction, police, lodge assistant, pensioners, and a wildlife tracker. The total number of focus group participants was 43 (Table 2).

**Table 2:** Demographics of focus group discussion participants.

Participants (FGD)		Value	SD
	Total Participants	43	
	*Men*	22	
	*Women*	21	
	Mean age	36	13.5
	*Men*	38	12.7
	*Women*	34	11.6
	Mean household size (individuals)	6	2.7
	Mean number of adults per household	2.4	1.25
	Mean number of children per household	3.4	2.38
**Highest Level of Education Completed**	** **		
	No formal education	8	
	Grade 8	1	
	Grade 11	4	
	Grade 12	28	
**Employment**			
	Unemployed/Pensioner	29	
	Community Caregiver	7	
	Other Employment (Construction, Chef, Lodge Assistant, Tracker, self-employed)	6	
	Not Specified	1	

### Observations

#### Human- and household-related tasks

For women, most household tasks were observed in the mornings in the verandas and outdoor kitchens. These tasks, mostly performed by the female head-of-household, included cooking and food preparation, cleaning, sweeping, collecting water, gardening, collecting wood, and taking care of household children. Afternoon tasks included food preparation, washing dishes, cleaning and sweeping, resting, and doing laundry. Men were less commonly observed within the households during the observation period. However, men who were observed in the morning were seen going to work, taking cattle to the bush, feeding domestic animals like cattle, pigs, and chicken, and collecting water. In the afternoon, men were observed resting, eating, running errands, or performing general maintenance/repair projects around the home. Men were also observed taking waste to dump sites in the bush or burying it in the ground of the household property. It was explained during the observations that children were attending school or initiation events during observation periods; it would not be typical for children to be at home unless during the holidays.

Household tasks and hygiene behaviors were mostly observed being performed by the female head of household. The women observed in the households spent most of their time in afternoon and evening in the outdoor areas of their properties while completing these tasks. Women in one village (Utha) were seen needing to spend more time, 3–4 hours daily, in the bush to collect water and wash clothes near the water source compared to the other villages where water was available directly or closer to the home. Men were also observed to have contact with bush areas when taking out trash and herding cattle for grazing.

#### Animals and environment

Locations and proximity of animal enclosures to the household areas were noted. The animal pens or enclosures, called a “kraal” in Shangaan local language, were located on the edges of properties but were often still close to cooking and cleaning areas where the family, and especially women, were observed to spend most of the daylight hours. These kraals were made of natural tree branches and trunks that were staked into the ground forming a natural fence. Kraals were observed to house cattle, goats, and swine. Chickens were either free-roaming or housed in wire pens, while dogs and cats were free-roaming. Households that did not own animals were still in proximity to outdoor animal enclosures on the bordering properties of neighbors Human-domestic animal interactions observed included chicken and goat feeding, herding cattle out to the bush in the morning for grazing and collecting them in evening. Goats, pigs, chicken, and cattle were kept in these kraals, but free-roaming animals, often coming to the property from outside the home, were also observed. In addition to domestic animals contacting the household from other parts of the community, individuals in one household observed described conflict in the community due to wild animals from a bordering game reserve killing their domestic animals. Dogs were not observed in separate locations and could be seen throughout the outdoor household areas or free roaming in the community. All members of the household including older generations were observed partaking in animal care and feeding.

**Fig 5:**
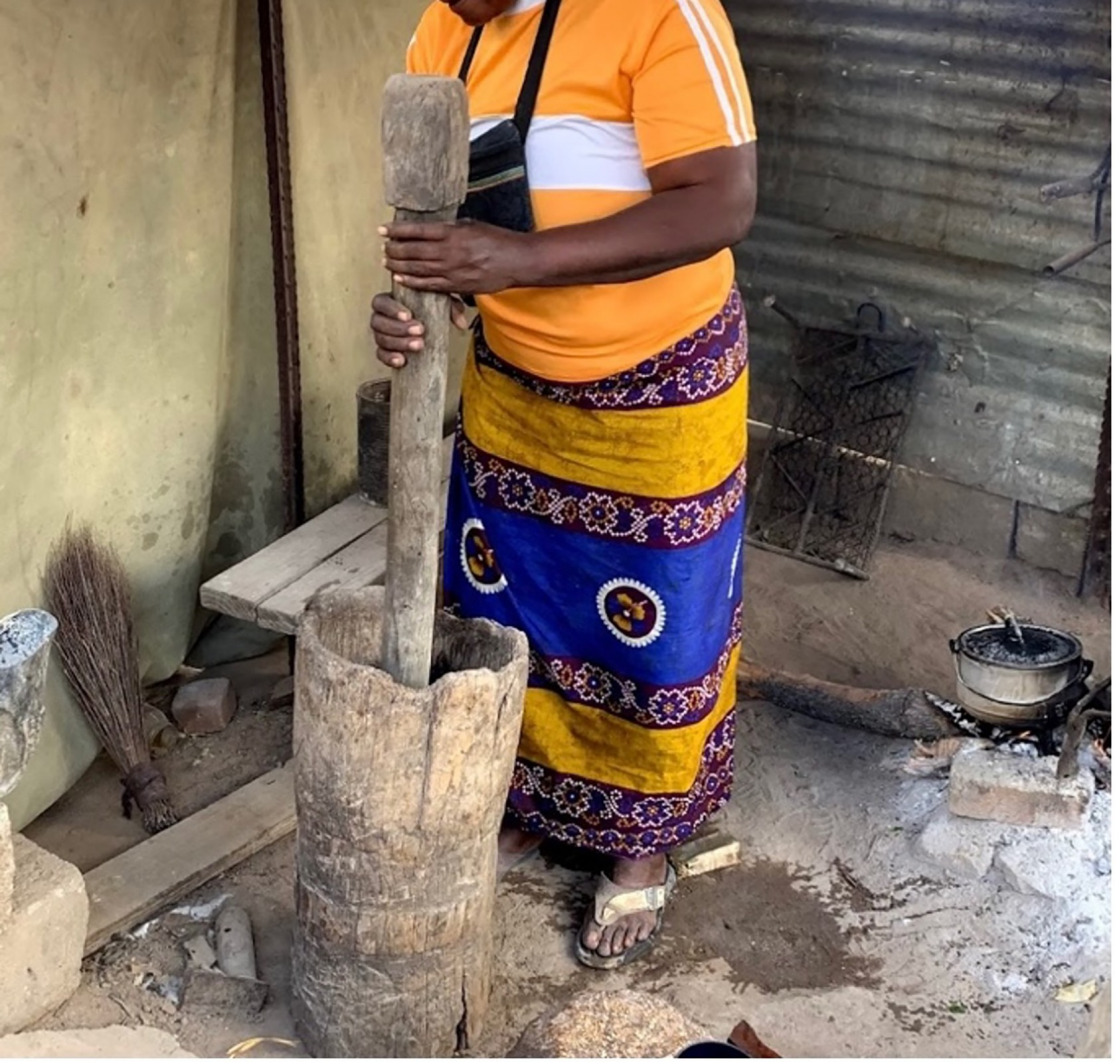
A woman cooking in a home kitchen with typically-observed attire.

Active prevention or removal of ticks and mosquitoes was not observed. Women were observed wearing typical clothing at home with shoes (Fig 5). This attire included a shirt, long skirt, and sandals. Those working in the bush or with cattle were seen wearing coveralls and shoes, though variations with people wearing no coveralls or open-toe shoes were also noted in observations. In addition to water retrieval, water storage in households was also noted. Water for cooking is stored in jerry cans, while water for other purposes such as washing is stored in open basins or in large plastic water tanks (“JoJo tanks”).

### Focus groups

#### Daily routines

Focus group participants were asked to describe their daily routines, including primary activities in the morning, afternoon, and evening. Table 3 summarizes the daily routines of both men and women. Women described that their morning activities included cooking (3 mentions), cleaning the house and yard (11 mentions), fetching wood and water (4), and preparing kids for school and getting ready for work (1 mention). In the rainy season, women also plough the fields in the morning (4 mentions). The rainy season occurs during the summer, November through to April, with the dry season occurring during the winter, May-October [21, 24]. Men described their morning routine including home improvement work (5 mentions), gardening/watering (5 mentions), collecting and chopping wood (4 mentions), drinking alcohol (2 mentions), cleaning (2 mentions), cooking (1 mention), exercising (1 mention), attending church (1 mentions), and doing laundry (1 mention). Men also described partaking in animal care in the morning for donkeys, cattle, goats, and chickens (4 mentions). In the afternoon, women’s routines included some of the same activities as during the morning in addition to watching TV or reading (6 mentions), cooking (3 mentions), bathing themselves and their children (4 mentions), going to the market (1 mentions), and grinding nuts (1 mentions). Men described “drinking” (14 mentions), “cleaning” (2 mentions), “fencing/painting/welding” (5 mentions), “don’t do anything/ “staying home” (2 mentions), and other activities including studying, soccer, and road work (3 mentions). Men in the focus groups also described feedings pigs and chickens in the afternoon (1 mention) and helping at the local school (1 mention).

**Table 3:** Daily routines of women and men reported in focus groups respectively by gender.

	Women		Men	
	Activity	Mentions	Activity	Mentions
AM	Cleaning	11	Home improvement	5
	Ploughing fields (Rainy season)	4	Gardening/watering crops	5
	Fetching wood/water	4	Wood collection	4
	Cooking	3	Livestock care	4
	Getting kids prepared for school	1	Cleaning/Cooking	4
Mid-day	TV/Reading	6	Consuming alcohol	14
	Cooking	3	Cleaning	2
	Bathing	4	Home improvement	5
	Market	1	Nothing/other	5
	Grinding nuts	1	Feeding chickens/pigs	1
PM	TV	3	Drinking	2
	Church	1	TV	2
	Helping children	1	Collecting cattle	2
	Braaing/cooking	1	Helping children	1
	Bathing	1	Feeding dogs/animals	1
	Sleeping	1	Playing games	1

In the evening, women described watching TV (3 mentions), going to church (1 mention), helping children with homework (1 mention), sleeping (1 mention), “braaiing” (cooking over fire) the head and feet of chicken (1 mention), and bathing themselves and bathing children (2 mentions). Men listed activities including drinking (2 mentions), watching TV (2 mention), collecting cattle (2 mention), staying with kids (1 mention), feeding dogs (1 mention), and playing games in the evening (1 mention). Children’s roles and activities explained in the focus groups included “letting the goats out to grazing” (2 mentions), fetching water, attending school, eating, and playing. To ascertain community perceptions of gender roles, we asked women in focus groups to state what activities men participated in in the community, and for men to state what activities women participated in the community. One male focus group participant describes, “my wife is busy reading books, the kids [are] also reading books” while another male participant describes “my wife is cooking, the kids are playing, all they do is play.” Men also described women grinding corn in the summer, cooking, washing, and cleaning the house. Women discussed that men collect firewood and work on fencing, go into the bush, build houses, or are at work or work in Johannesburg. One participant described that most men in her community are unemployed.

We also sought to understand seasonal differences in household tasks and activities to better understand seasonality as a factor for zoonotic disease exposure. Focus group participants mentioned that crop planting and plowing occurs in the summer (4 mentions). One participant noted that in the summer they follow the cattle into the bush all day because the cattle eat people’s crops. In the winter, participants stated that men take children to initiation schools, a traditional practice in many communities in rural South Africa that involves cultural education and coming of age ceremonies for boys and girls [44].

#### Hygiene behaviors

Discussion about hygiene behavior centered on washing and storing dishes, sweeping the yard, and hand-washing behaviors. Four participants in the women’s groups noted that dishes are stored inside the home to avoid dogs and goats from licking them. As one female participant notes, “I sleep where the dishes are, I don’t want the dogs to lick them.” Other discussion around hygiene with animals involved wearing gloves to pull calves being birthed (male participant), and cooking inside the home to avoid rats and cockroaches. Five female participants mentioned cleaning refuse of cattle or goats as a reason for cleaning the yard. Six participants, both male and female, mentioned sweeping their yards for cleanliness of the home and prevention of disease. One female participant noted, “To stay in a clean place, it gives a healthy life.” When asked if handwashing was common, all participants agreed they washed their hands during the day. Both male and female participants noted washing their hands with soap and water after using the toilet (4 mentions), removing ticks (2 mentions), before or after eating (3 mentions), when preparing food (1 mention), and after activities such as spraying the cattle (1 mention, male participant) and handling chemicals, rubbish, or something else dirty (1 mention, male participant).

#### Animal care

From the discussion, both men and women participate in animal care duties. There were six mentions of women caring for animals and 13 mentions of men caring for animals from the FGDs. Confirming the observation data, women primarily cared for poultry and small livestock, while men mainly tended to cattle. However, there were mentions of women also caring for cattle and sharing those duties with the males in their households (2 mentions). It was confirmed from the observation that men typically take the cattle to the bush to graze in the morning and collect them in the late afternoon. There were three total mentions of participants who have hired cattle caretakers, who are male. One female participant did mention her family hired a caretaker to take cattle into the bush all day. There were five mentions of male participants pulling calves when they are struggling to give birth, with two participants mentioning they do not help with birthing calves and call for the community vet or the local animal health technicians to assist with these. The FGD positively confirmed the observations that men typically take cattle out to the bush to graze early in the morning around 7am and bring them back 4pm (6 mentions). Some noted they do not bring back the animals everyday (3 mentions). Slaughter is also conducted at the household. Confirming previous study results, women primarily slaughter poultry only (6 mentions), while men slaughter goats and cattle (5 mentions) because as described by one participant, “[It’s] very difficult to slaughter cattle and goat, difficult because they need a man’s power.” However, some personal preference can be noted here, as two women in the focus groups discussed that they did not slaughter any animals in the home. One woman stated, “I just buy [meat] somewhere when I want meat. When I eat that meat, I think about that animal’s life.”

#### Animal use and ownership

There was consensus among men and women about the significance of animals in the community and in describing use and ownership. When asked to describe the use and purpose, or value of animals in the community, participants described dogs as “protection” and “security” (4 mentions). Cattle, goats, and poultry are used for food production, to sell, and for financial security (16 mentions). One participant noted that cattle signify wealth and financial security in the community. Participants also described a sentimental or cultural value to owning animals (5 mentions). One male participant described, “I love the animals, it’s our nature to have animals.” Another male participant also detailed how goats and cattle have cultural value for use in ceremonies and graduations.

#### Human-animal conflict

Human-wild animal conflict in the community points to the increasing interactions at the wildlife-livestock-human interface. These conflicts occur both with wild and domesticated animals. One male participant noted, “People are complaining about wild animals, they eat our cattle; hyenas are eating our cattle; some are bitten by stray dogs.” These mentions of human-wildlife conflict indicate that contact between domestic animals and wildlife is occurring, thus presenting opportunities for transmission of pathogens (direct contact), contamination of the environment (indirect contact), and movement of vectors into human-dominated landscapes (vector-borne).

#### Water

Water access was noted as a major issue in the community. Water collection was a primarily womens’ task based on both observations and FGDs. Focus group participants noted that if water is supplied to the home, it is available 1–3 times a day (3 mentions). On other occasions where water is not available at the home, water is sourced from community boreholes (3 mentions), the river (2 mentions), buying water in the near town (2 mentions) or from local schools (1 mention). The major source of fresh water for all communities in the area is the Injaka Dam, approximately 55 kilometers from the town of Hluvukani. Water is typically in large water tanks (JoJo’s), buckets, jerry cans, and basins. Female participants described using wheelbarrows to collect water from communal boreholes or tanks (2), which can be a laborious task. One participant noted she sometimes finds rats in the jerry cans used for kitchen storage of water. The issues of water access also impact animal species. One participant noted, “There is no water in the riverbed, the animals are getting water in the house.” Another participant described, “I pour water for the animals in a basin, there is none in the dam.” Because of drought and water shortages, raising and tending animals has become more difficult. As one participant states “During drought, animals don’t have food, thin animals are hard to sell at butcheries.” One participant even described feeding soapy water to her goats as to not waste the water used for washing.

#### Vectors of infectious disease

Participants were asked to describe their interactions with potential vectors of infectious diseases (e.g., ticks, mosquitoes). Both men and women described seeing ticks in the winter (June to August) and mosquitoes in the summer (December-March), though both vectors were noted as present in both seasons. Significant gender differences were not noted in FGDs surrounding attitudes and perspectives on vectors. Several strategies for bite prevention included wearing protective clothing (3 mentions), using pest repellent products like Peaceful Sleep and Doom (7 mentions), using mosquito nets (4 mentions), checking their own body for ticks or asking someone to check their body (5 mentions), and burning mosquito coils or cow dung in the home (5 mentions). Five participants described removing ticks from themselves with their nails (5 mentions); no other removal methods were mentioned. Tick removal was also discussed for animals. Methods mentioned included using lye, car oil, or insect repellent to remove ticks from animals like goats and dogs (3 mentions). It was also mentioned that dogs are taken to the cattle dip tanks (2 mentions). The discussion revealed concern for tick infestations for animals. One participant noted, “Ticks are showing between the nails and the goats hurt.” Another participant followed, “Goats will limp and don’t take those [ticks] off themselves because it is painful. We are scared they will have a fever if not removed.” There is a perception of animal illness and discomfort from ticks. As later mentioned by another participant, “the animals might die because of ticks.” There was also the mention of potential human impact of tick bites, as one participant mentioned, “Ticks are irritating, it can give you diseases. We don’t want them because it can spread diseases.” One male participant also described that “ticks are irritating us, [they] can give you diseases, we don’t want them because it can spread diseases.”

#### Additional community concerns

One primary concern discussed was the overgrazing of wild bush areas surrounding the communities. One participant stated, “The grazing area does not have enough grass, so animals are starving.” This sentiment was echoed by two other participants. Focus groups responses indicate that the community does have concerns about disease spread from animals and vectors and among animal populations. For example, one participant posed a question when asked if they had any concerns for their community of, “How can we stay together with our dogs when they maybe bite, and they are sharing some diseases?” The community members are aware of potential disease issues but may want more information on how to prevent disease spread and protect themselves and their animals.

### Data triangulation

Findings from both the focus groups and observations supported each other in several instances. For example, it was confirmed that daily activities for women included cooking, cleaning the yard, fetching water, washing, and childcare; daily activities of men included animal care, collecting firewood, going to work or working on the house, and gardening. We confirmed who takes care of animals, with women more frequently tending to smaller livestock such as goats and poultry, and men tending to cattle and pigs. Additionally, we confirmed our observation of where animals were located both in the house and when they are grazing, with poultry, dogs, and cats typically free-roaming, and pigs, goats, and cattle kept in kraals on the owner’s property. When grazing, goats and cattle were able to travel to the bush near the house.

There were instances in which we observed personal perspective in the focus group differing from the larger consensus of observations. For example, there were differences reflecting personal preference of lifestyle in the daily activities that was different than what was observed within that gender group. For example, a female focus group participant stated she does not slaughter chickens herself, while the others did. Prominent differences in gender roles between the observations and focus group responses were not observed. We noted gender differences for household tasks, animal care duties, and environmental exposure. Emergent themes included decreased water and grazing land availability in the community affecting both men and women, as water collection and cattle grazing now required more time spent in the bush and increased potential vector-borne disease exposure for both genders. From observations, it was noted that men wore covered protective work clothes (such as long trousers and closed-toe shoes) more commonly than women did; women did not often wear these for household duties including water collection in the bush. Table 4 summarizes the themes derived from the focus groups and interviews.

**Table 4:** Themes derived from observations and focus group discussions.

Name	Number of References
Domestic Animal	201
Animal Locations	60
Animal Care	50
Animal Use	20
Animal Contact	19
Buying and selling	17
Animal Conflict	16
Men Care	13
Women care	6
Environment	131
Water	79
Vector Exposure	16
Waste	14
Built Environment	11
Wildlife	4
Overgrazing Bush	4
Agriculture	2
Natural Environment	1
Person	99
Mother or Female Head of Household	36
Daughter	28
Father or Male Head of Household	13
Grandmother	9
Son	8
Hired animal caretaker	3
Grandfather	2
Location of Activities	34
Yard	10
kraal	10
Bush	6
kitchen	6
Other Home	2
Human	335
Hygiene	79
Food prep	45
Daily Routine	33
Division of Labor	30
ID risk	10
Clothing	8
Customs	7
Community concerns	7
Caregiving	6

## Discussion

**Fig 6:**
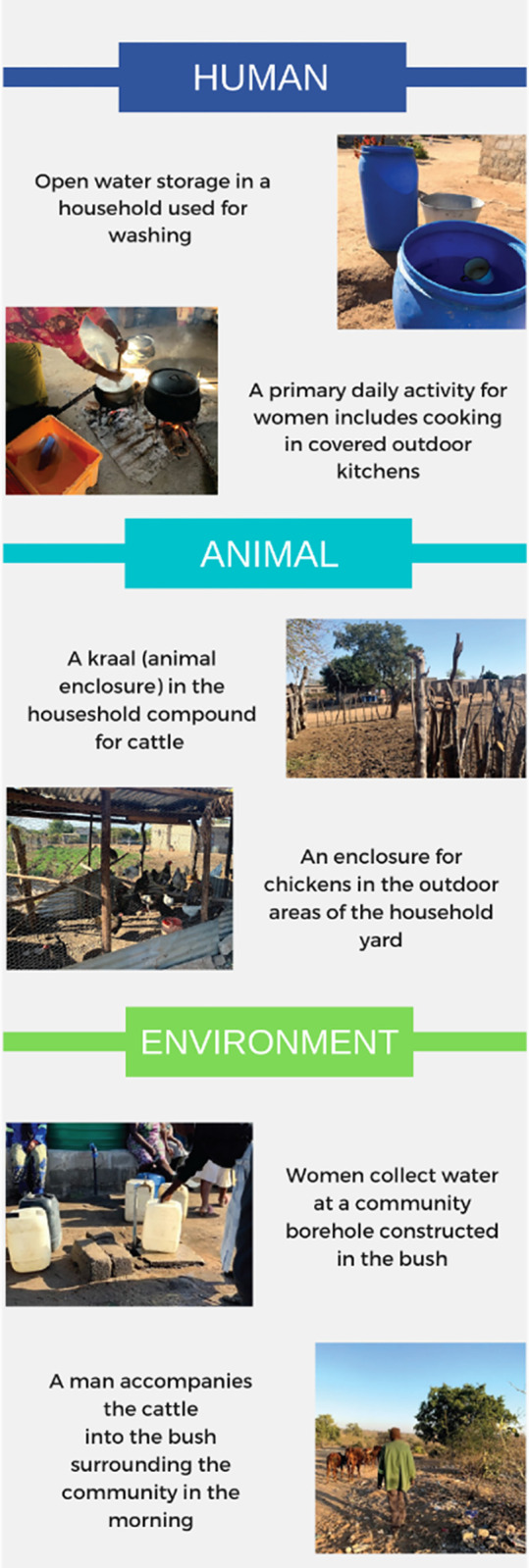
Images of human-animal-environment interactions observed in the Mnisi community, South Africa.

Through the analysis of data generated by household observations and FGDs, this study has identified daily livelihood tasks and associated gender roles in the context of zoonotic and vector-borne disease exposure in the rural Mnisi community of Mpumalanga, South Africa (Fig 6). Certain tasks that may indicate an exposure differential for infectious disease are highlighted. For example, household, animal, and environmental exposures differed by gender, though potential existed uniquely in both gender groups. Protective clothing and activities within the bush were also found to differ between men and women.

### Gendered tasks

Daily life is largely gendered in the community, with clear roles within households and communities for livelihood tasks, animal care, childcare, and hygiene behaviors. Women are primarily responsible for household tasks including cooking, cleaning, sweeping, washing dishes and clothes, and caring for children. Women are primarily responsible for household hygiene tasks. Therefore, interventions that focus on hygiene behaviors at the household level (e.g., water sanitation and hygiene–WASH) should be targeted towards women and include a gendered perspective [45]. Interventions for men’s hygiene behaviors can focus on their specific exposures working outside the home. Women also tend to small livestock. Except for women who work outside the house, women observed at homes spent most of their day at home but still are tasked with responsibilities requiring environmental interactions such as water and firewood collection and plowing fields in the summer season. Men’s activities in the community included working outside the home or within the community. Men still were responsible for household tasks such as collecting water and firewood, building homes, waste removal, tending crops, and caring for cattle. Regarding potential infectious and zoonotic pathogen exposure from animals, vectors, and the environment, we were able to ascertain differing potential exposures by gender. Men are primarily caretakers of cattle in the community and are responsible for slaughtering cattle. Many households that own cattle, however, do hire a caretaker to follow their cattle in the bush. Therefore, we believe it best to consider men to have higher possible exposure by direct and indirect contact with cattle and their vectors, with those men especially exposed being those who are hired to take care of cattle (e.g., herders). These caretakers were also described to stay extended periods of time in the bush, therefore increasing their duration and frequency of exposure to vectors and infectious pathogens potentially spread by wildlife. In this region, priority bacterial pathogens of those exposed to cattle include *Bartonella* spp., spotted fever group (SFG) *Rickettsia* spp., *Coxiella burnetii*, and *Leptospira* spp. [26]. The use of cattle caretakers may also pose an interesting question about the role of socioeconomic status and risk of disease. Owning cattle and hiring a caretaker represents a degree of wealth in the community and may also pose a differential in potential environmental exposure.

### Water

The data describe women primarily being responsible for water collection, storage, and management in the household. Though various sources of water were described in the three villages in the study, it was noted throughout that reliable water access for both humans and animals are primary issues in the community. As was seen in the study, community boreholes, often in the bush, are still the primary means of water access. In relation to potential infectious disease exposure, women carry the burden of water collection responsibilities, which can potentially increase their time spent in the bush exposed to disease vectors and zoonotic pathogens. This study also identified community-level water variability, suggesting village of residence could also play an important role in environmental exposures and sanitation and hygiene Examining gender roles in water collection, storage, and use can further answer to what extent water scarcity and gender impact both animal and human health in the community. Water was a theme explored across villages. Water sources were varied across the three villages, with Athol and Utha relying mainly on boreholes and purchased water, and Gottenburg having more water access in their homes. This correlates with previous surveys of the community, where Utha community members perceived water reliability least favorably and Gottenburg residents perceived water reliability most favorably [24]. The open water basins could serve as additional risk factor for mosquito proliferation at the household level, which water scarcity may contribute to difficulties in proper sanitation availability. Unhealthy animals caused by resource scarcity from environmental shifts may pose a larger risk for infectious disease transmission and mortality to both humans and animals [46].With current climate and agriculture trends, livestock production will be increasingly challenged by land and water scarcity, threatening food security and nutrition [47].

### Land use

Overgrazing and degradation of the wild bush areas surrounding the communities was a major concern from the FGDs for both gender groups. With less viable grazing areas, cattle spend a longer time in the bush, and require attendants to also go deeper and farther to retrieve cattle. Much like water access, this is critical area of the human-animal-wildlife interface that should be further examined to understand its impact on zoonotic disease transmission dynamics, including how gender is involved due to the gendered nature of tasks requiring bush access. Increasing wildlife-human conflicts noted in the community also pose another additional zoonotic exposure route. The environmental impacts of landscape changes and natural resource scarcity can have implications on changing behaviors and roles within the community, and therefore can potentially be concern for increasing zoonotic disease transmission. Both humans and animals in this context share the same environmental exposures. Effectively addressing this shared environmental risk would expand control measures to include managing landscape and land-use change to benefit both animal and human populations [44].

### Perception of disease risk

Through FGDs we were able to understand that there is a perception of disease risk from animals and the environment to residents in the community; however, knowledge of specific transmission mechanisms and prevention behaviors may not be as widespread. A desire for this type of training, based on their unique risk factors and behaviors, was expressed by the community. Previous research in the Mnisi community found a statistically significant gender differential in those presenting for acute febrile illness with evidence of zoonotic pathogen exposure [25]. This study helps describe the risk environment for infectious zoonotic diseases in the region, which can also help in further tailoring programming by gender in the community that may also be influenced by larger environmental factors such as water scarcity and degradation of natural bush areas surrounding the Mnisi community.

Using ethnographic methods, this study was able to confirm and contextualize the gender-differentiated tasks that have been previously described in this community [28]. Additionally, this study elucidated tasks women engage in daily, including water collection from the bush for both drinking and washing, firewood collection, plowing the fields, and smaller domestic animal care. We hypothesize these tasks serve as potential transmission pathways for common zoonoses (e.g., spotted fever rickettsiosis) that are more likely in women [25]. As such, protective clothing and prevention measures should be explored that still fit within the cultural expectation and norms of the community, as women were more commonly seen wearing open-toed sandals and skirts rather than boots and coveralls. This study also provides context for various household member roles. These contexts should be considered in developing One Health educational programming targeted towards certain groups and villages in the Mnisi community. Participants expressed desire to learn more about disease topics relating to their animals and the environment, which should also be considered for further development of community outreach initiatives. Comparing men to women in this study, we see that they do have differing roles, but there are opportunities for zoonotic pathogen exposure for both men and women due to the nature of tasks undertaken by both genders.

Framing the study in ecosocial theory, we see how individual embodiment of gender, and therefore gender roles, can influences health outcomes and patterns of zoonotic disease prevalence. The disparity may stem from differing livelihood activities, caused by differing embodied social states of gender, and therefore differing potential environemtnal interactions and exposures to pathogens. Integrating this theory into future work, we can continue to understand the web of causation that may contribute to zoonotic disease spread not only considering animal and the environment, but also the social and cultural factors that inluences human behavior [36].

### Community shifts

With the increased endangerment of natural resources in the Mnisi community and the greater Bushbuckridge area, the economy is shifting away from subsistence practices and towards outside land-based income sources [48]. Environmental conditions such as drought and lack of grazing areas make raising livestock even more difficult. The erosion of socioecological systems in Bushbuckridge has direct implications on environment and sustainable resources [49]. Factoring in infectious disease both to animals and humans, it will be important to examine the future of subsistence farming in these communities and how it will impact the interactions between humans, animals, and the environment. Though there is an overall downward trend of ownership in the area, our study highlights cultural aspects of animal ownership that continue to tie the Mnisi community to animal rearing and care. While there is still consistent daily contact and exposure to wildlife and livestock, measures should be taken to ensure those who are still dependent on livestock will need additional consideration of the risk of infectious zoonotic pathogens to their families and homes, especially in the context of gender, where women may not be readily factored into these issues. This information informs future research and policy to mitigate infectious disease with the necessary background information to integrate the appropriate cultural context of the community to achieve the desired outcomes more effectively.

### Strengths and limitations

By directly observing community members perform daily tasks, this study provides a greater social and environmental context for activities happening within the household rather than relying solely on participant verbal description. Researchers could visualize infrastructure, note various activities occurring concurrently, and observe locations and proximity of animals in the context of these activities. Observations also helped ascertain the emic perspective of the study participants. A drawback of this method is the depth of information needed. Due to time and budget constraints of the study, observations occurred at a single point in time and may not have included each household member. Follow-up ethnographic research should consider an extended stay within the community (e.g., 6–12 months); this would allow for direct observation of all household members fulfilling their roles during multiple seasons. Additionally, actual disease prevalence or risk specifically associated with these behaviors and livelihood activities was not assessed and can be a future research objective now that the behaviors are described and contextualized.

A strength of using focus groups was that we were able to capture men’s and women’s perspectives separately. This was relevant not only for analysis of the data through a gender lens, but also to provide additional space for men and women to share their perspectives more openly [50]. Additionally, with focus groups we were able to include several more members of the community to provide input for the research, and the open-ended nature of the questions provide a greater insight using the participants’ own words [51]. Drawbacks of FGDs include potential bias from the presence of the moderators, the requirement of a translator live during the FGD, and the focus group only representing a subset of the community at the given time. Translation of the focus groups from Shangaan to English may also impart challenges, particularly with regard to the explanation of concepts related to health, illness, and disease and the way it is specifically referenced in this community. For example, scientific and medical terms may not exist in another culture or language, making an accurate and precise translation more difficult. Having in-depth analysis of the focus groups from a Shangaan speaker in the future would increase confidence in the interpretations of the discussions and participant responses and additional richness to the context of the responses.

### Future directions

A further implication of this study is to provide a context for which to focus future zoonotic disease prevention efforts. For example, a diagnostic scheme for local clinics can be developed for those presenting with signs of infection from a zoonotic or vector-borne disease to best address unique gender and environmental exposures that may make certain disease exposures more likely. Additionally, if further One Health training programs are developed in the area, this study can help tailor trainings to members of the community based on the transmission dynamics of specific diseases like Tick Bite fever, leptospirosis, and Q fever. These trainings could be created to be inclusive of the differing lived experiences and exposure of children, women, men, and those who work in settings where they may be at increased risk, such as cattle caretakers. Diarrheal disease is a major cause of infant mortality globally and, while it is likely also responsible for morbidity among adults/children in the Mnisi community area, this outcome has not been determined as it relates to zoonotic pathogens and, thus, was not the focus of this study. Future studies can factor in the prevalence of diarrheal disease in children in the community to better understand the zoonotic component of diarrheal diseases in rural South Africa. Future survey methods can also be improved to increase opportunity for direct perspective and observations of community members and their environment to show more depth of how these daily tasks, activities, and exposures occur. This study highlights the necessity of these qualitative methods for providing context for survey results and quantitative epidemiologic data.

This work could be expanded by including further and extended participant observation throughout the year and in varying seasons. This observation could focus on cattle caretakers who spend all day in the bush, children’s education on zoonotic and vector-borne disease, tracking distances cattle go daily and tick densities in these areas, and time allocation analysis for certain activities. Additionally, comparing the daily activities recorded to updated epidemiologic and diagnostic data from the community will further help to assess the impact of shifting ecological, economic, and social changes on infectious disease in the community. Future research should also include children in both FGDs and observations to better understand their unique roles, potential exposures, and high risk for diarrheal diseases In context of the COVID-19 pandemic, it will be necessary to examine how roles and responsibilities in the community have shifted or stayed the same, especially in relation to subsistence practices (e.g., animal rearing, water collection) and childcare (schooling issues). Additionally, it would be necessary to factor in any changes in hygiene and social distancing practices for COVID-19 prevention, dip tank operation, and other animal co-mingling. The gender disparities evidenced during the COVID-19 pandemic globally also highlight the need for taking a gender lens to emerging infectious disease topics [52]. This gender lens can be used to develop further educational materials, One Health training programs, animal health and veterinary support programs, and policy changes that address infectious disease prevention. Taking a gendered approach in One Health is necessary to fully understand the depth of impact of social factors on infectious disease exposure and risk.

## Supporting information

S1 File Focus group discussion guide. (DOCX)
