# Supplementary material for: Gender and zoonotic pathogen exposure pathways in a resource-limited community, Mpumalanga, South Africa: A qualitative analysis
Source: PLOS Glob Public Health. 2023 Jun 5;3(6):e0001167. doi: 10.1371/journal.pgph.0001167 (PMC10241411; doi:10.1371/journal.pgph.0001167)
Supplement: S1 File — (DOCX) [file pgph.0001167.s001.docx]

**Supporting information**

**S1 File. Focus Group Discussion Guide**

Sample Size and Selection: Two focus groups will be conducted in each village, one with men and one with men. With three villages selected to participate, we will conduct 6 total focus groups, each consisting of 5-7 people.

Introductory Script: Thank you for joining our group today to discuss daily activities and health of men and women in your community. We expect this discussion to last about an hour. You will be asked questions that will allow researchers to gain a better understanding of what daily life is like in your community. We will not be recording your name, and no one will be able to connect you to the responses you give. This will help protect your privacy. We ask that you do not share information about other participants outside this group. There are no right or wrong answers to the questions we will be asking, so please feel free to share your own opinions and experiences openly.

**A) Introductions: To start, please tell me a little bit about yourself and your household:**

1. What is your age?
2. How much schooling do you have?
3. Who lives in your household with you? (How many people total, how many adults, children?)
4. Do you have a job outside the home? Describe your job.

**B) Describing a typical day**

1. What are activities and tasks typically done in the morning (wake up to 12pm?)
2. What tasks do you do in the afternoon?
3. What tasks or activities do you do in the evening?

Describe how each of those tasks or activities are performed and how much time is spent in each activity every day (hours, etc). (For women, focus on human and household tasks such as food preparation, water collection, cleaning; for men, focus on animal husbandry and wildlife tasks such as herding, slaughtering, and hunting.)

1. What is the source of your water?
2. How reliable is your water source?
3. Where do you get water if your primary source is not available?
4. What do you use the water for?
5. Do you treat or filter your water?
6. How and where do you store water?
7. Where do your animals get water?
8. How often/when do you cook?
9. How do you handle meat before cooking?
10. Where do you store pots/plates/cooking tools? Why?
11. Why do you sweep your yard?
12. From whom and when did you learn how to do these household tasks or activities?
13. Ask women to describe a typical day for men/children; ask men to describe a typical day for women/children. Do these tasks differ by season/time of year? If yes, how?

**4) Outdoor Activity and Disease Prevention**

1. When you spend time/work outside, what kind of clothing/footwear do you wear?
2. How frequently do you get insect bites (e.g., ticks, mosquitoes) – what about during the dry vs. wet season (winter vs. summer)?
3. Do you try to prevent insect bites (e.g., ticks, mosquitoes) while spending time outside/inside the home? If yes, how?
4. Do you check your body for ticks? If yes, how?
5. How do you remove ticks if you find them on yourself? Do you try to remove ticks from your animals? If yes, how?
6. How do you wash your hands? When do you wash your hands during the day?

**D) Animals**

1. Do you have animals and what kind do you own? (Cattle, goats, chickens, dogs, cats, pigs, donkey, etc.)?
2. Who takes care of the animals? Describe what tasks you typically perform to care for your animals.
3. Where do you keep the animals? Do they roam freely or are allowed to graze freely? If they roam, where do they roam and when do you let them out and collect them back?
4. Do any animals come inside the home (living areas)?
5. Do other people’s animals enter your property? If yes, how often? Do you try to keep animals out of your living areas?
6. What is the purpose or use of your animals? How often do you buy new animals? Do you sell any animals and if so to who?
7. Do you slaughter animals (chickens, goats, cows, pigs) in your home? Who slaughters the animals and how?
8. Do you help your animals when they are giving birth? If yes, how?
9. Any final thoughts or questions you would like to share about life, health, or owning animals in your community?
